# Neuromotor mechanisms of successful football penalty kicking: an EEG pilot study

**DOI:** 10.3389/fpsyg.2025.1452443

**Published:** 2025-05-19

**Authors:** Dan Li, Hatem Elbanna, Fang-Yi Lin, Chieh-Ju Lu, Li-Ju Chen, Guo Lu, Chien-Lin Yu, Kuo Pin Wang

**Affiliations:** ^1^College of Leisure and Social Sports, Jilin Sport University, Changchun, China; ^2^Department of Sports Psychology, Faculty of Sport Science, Mansoura University, Mansoura, Egypt; ^3^Center for Cognitive Interaction Technology (CITEC), Bielefeld University, Bielefeld, Germany; ^4^The Master Program of Sport Facility and Health Promotion, National Taiwan University, Taipei, Taiwan; ^5^International Football Education School, Jilin Agricultural University, Changchun, China; ^6^Department of Physical Education and Sport Science, National Taiwan Normal University, Taipei, Taiwan

**Keywords:** psychomotor efficiency, neural efficiency, electroencephalography (EEG), motor preparation, cognitive-motor performance, attentional allocation

## Abstract

**Introduction:**

Understanding the neuromotor processes underlying successful and unsuccessful performance in lower limb movements, such as football kicking, is essential for athletes. However, further investigation is needed to determine whether lower activation of the prefrontal and central cortices, which are associated with motor programming, such as motor planning and motor control, corresponds to higher degrees of psychomotor performance in a difficult task. To address this issue, this within-subject pilot study aimed to investigate neuromotor processes in skilled football players performing penalty kicks under a difficult condition.

**Methods:**

Ten skilled football players were recruited and asked to perform 30 kicks under a difficult condition where the target size was adjusted to maintain a kicking success rate between 40 and 60% for each player while we recorded EEG data during motor preparation. Afterwards, EEG power in the 8–13 Hz frequency band was analyzed at frontal (Fz) and central (Cz) regions before action.

**Results:**

The main result indicated that successful penalty kicks were associated with lower 8-13 Hz power at frontal and central regions before action, suggesting efficient neural activation for motor planning and control during motor preparation.

**Discussion:**

These findings support the model of attention allocation and the psychomotor efficiency hypothesis, aligning with similar neuromotor processes observed in golf studies. Overall, this study highlights the critical role of motor planning and control in successful athletic performance.

## 1 Introduction

In sports, the ability to effectively adapt to varying levels of task difficulty is crucial for an athlete's performance. As tasks become more challenging, athletes must engage in advanced motor programming processes, such as motor planning and motor control (Cooke et al., 2015; Wang et al., 2022). These processes often involve heightened attention focused on specific actions. For instance, excessive reliance on explicit knowledge of movement mechanics can interfere with the smoothness and automaticity of movements, leading to performance decrements (Maxwell et al., 2000). In football, this phenomenon is exemplified when a player overanalyzes their kicking technique during a crucial penalty kick, resulting in a less accurate shot (Slutter et al., 2021). Difficult tasks have the potential to disrupt the optimal, automatic, and flow-like mental states observed in elite athletes (Bertollo et al., 2016). However, not all challenging tasks necessarily lead to performance decline. Thus, investigating the neuromotor processes that support an adaptive mental state in response to increased task difficulty is essential for understanding motor performance, particularly in lower limb movements, such as penalty kicking.

The model of attention allocation posits two essential characteristics: a filter and a fuel (Wickens and McCarley, 2008) to explain optimal performance. The filter characteristic involves the ability to selectively ignore task-irrelevant information, while the fuel characteristic enables the processing of task-relevant information (Wickens and McCarley, 2008). This model mirrors the psychomotor efficiency hypothesis (Hatfield, 2018; Wang et al., 2022), in which “psychomotor” refers to bodily movements regulated by psychological processes, emphasizing the coordination between cognitive mechanisms and motor execution, as well as the efficiency of neural and motor functions during task performance. From neuroscientific perspective, it explains the fundamental characteristics of superior motor performance: (1) the selective activation of crucial neural processes and (2) the suppression of irrelevant neural processes during motor preparation (Hatfield, 2018). Both the filter and fuel processes are pivotal for skilled football performance, especially under challenging conditions (Wickens and McCarley, 2008). For example, increased task difficulty demands higher mental effort (Cooke et al., 2015). Athletes who can effectively filter out irrelevant cues and efficiently process relevant information can maintain the optimal mental state for superior performance (Chen et al., 2022; Cooke et al., 2014; Wang et al., 2020). Accordingly, understanding how skilled football players adaptively allocate attention to motor programming during a challenging situation can reveal ecologically valid mental strategies, offering critical insights for enhancing athletic performance.

Previous studies have used electroencephalography (EEG) power analysis (Cooke et al., 2014; Wang et al., 2019) and event-related desynchronization (ERD)/synchronization (ERS) analysis (Babiloni et al., 2008) to test the psychomotor efficiency hypothesis and identify the neuromotor processes underlying successful and unsuccessful performances in challenging tasks. Specifically, ERD refers to a decrease in EEG power at certain brainwave frequencies during a specific event, which typically indicates increased cortical activation, meaning that the brain area is actively processing information (Pfurtscheller and Da Silva, 1999). Conversely, ERS refers to an increase in EEG power at specific brainwave frequencies during an event, usually reflecting a decrease in brain activity or an inhibited state in that brain area (Pfurtscheller and Da Silva, 1999). These changes are closely related to motor control and attention allocation, making them highly valuable for investigating motor performance (Babiloni et al., 2008; Wang et al., 2019). For example, previous studies adopted individual task difficulty, meaning each participant performed a task with a 40–60% success rate as a challenging condition and revealed more alpha (10–12 Hz) ERD (i.e., increased excitability) in the frontal and central areas (Babiloni et al., 2008), lower alpha (8–13 Hz) power (i.e., higher activation) in the frontal and central areas before successful performance in skilled golfers (Cooke et al., 2014; Wang et al., 2019). This reflected the effective allocation of attention to the core components of motor control, selection of task-relevant cues, and prevention of excessive reinvestment of attention on the action (Cooke et al., 2015; Wang et al., 2021). However, the above-described studies have a major limitation. Specifically, previous studies focused on upper limb motor movement in sports, such as golf putting. The findings from these studies may not be generalized to lower limb movement in sports, such as football kicking. Slutter et al. (2021) used functional near-infrared spectroscopy (fNIRS) to examine brain activation during penalty kicks under pressure. Their findings showed that higher activation of the prefrontal cortex (i.e., F3, Fz, and F4) was associated with missed penalties compared to scored penalties in a difficult condition. However, this result contrasts with previous studies (Babiloni et al., 2008; Cooke et al., 2014; Wang et al., 2019). Given that we do not know whether the inconsistent finding is because of a pressure manipulation or different types of movement (upper limb movement vs. lower limb movement), adopting a similar research design (EEG power analysis and a task difficulty) without pressure manipulation from previous golf studies could address this issue. By using a similar approach, we could determine whether the observed differences in brain activity are due to the type of motor skill or the pressure manipulation involved in the experiment. Importantly, it can provide a deep understanding of the neuromotor processes underlying successful and unsuccessful performances in lower limb movements, such as football kicking.

This pilot study aimed to specify the neural mechanisms underlying successful performance in a lower limb motor skill (i.e., football penalty) using EEG. Specifically, we examined neuromotor processes in the 2 s before movement to understand how attentional allocation adapts under a difficult task condition. Since lower 8–13 Hz power at frontal (F3, Fz, and F4) and central regions (C3, Cz, and C4) has been linked to successful performance in precision-based tasks (e.g., golf), we hypothesize that successful penalty kicks in skilled players will be associated with lower 8–13 Hz power in these regions compared to unsuccessful attempts under a difficult condition.

## 2 Materials and methods

### 2.1 Participants

Ten male skilled football players (mean age = 23.9 ± 2.28 years, mean height = 178.3 ± 5.16 cm, and mean training frequency = 3.5 ± 4.1 times per week) participated in this study. Players were classified as “skilled” based on their participation in regular annual regional-level tournaments in Germany for at least 5 years, along with their consistent training in competitive football. All of the recruited participants met the following criteria: (a) no history of neurological disease, (b) right-handed (Oldfield, 1971) and right-footed (Carey et al., 2009), and (c) not taking any medicine affecting the central nervous system or brain, (d) normal or corrected-to-normal vision, (e) normal visual selective attention by using Trail Making–A Test (Partington and Leiter, 1949), (f) provided informed consent to the research conditions as specified by the Research Ethics Committee of Bielefeld University.

### 2.2 Study design

This study employed a within-subject pilot study design to investigate the neuromotor processes underlying penalty kick performance in skilled football players. EEG data were recorded during motor preparation (2 s before action) to examine differences in neural activation between successful and unsuccessful penalty kicks under a difficult task condition.

### 2.3 Study measures

#### 2.3.1 Football penalty task

Participants in this study engaged in a football penalty task using a standard Size 5 football (diameter = 23 cm, circumference = 70 cm), aiming toward a football goal measuring 450 cm wide and 250 cm high, from a kicking distance of 1,100 cm. The target size was adjusted to achieve a kicking success rate between 40 and 60%. Initially, the target size was set at a square of 70 × 70 cm, and each participant performed five kicks. Adjustments were made based on the individual success rate: if the success rate was within the desired range (40–60%), the target size remained unchanged at 70 × 70 cm. If the success rate exceeded 60%, the target size was reduced to 60 × 60 cm, and an additional set of five kicks was performed to confirm consistency within the 40–60% range. Conversely, if the success rate fell below 40%, the target size was increased to 80 × 80 cm, and five more kicks were executed to achieve the optimal success rate. Once the appropriate target size ensuring a 40–60% success rate was determined, participants proceeded to perform a total of 30 kicks. The motor preparation period is defined as the time between being motionless in position and initiating the kick. Importantly, successful performance was defined as a trial in which the ball hit the target.

### 2.4 Subjective anxiety level

To control for potential confounding effects of anxiety, the individuals were asked to report a feeling of anxiety level with a visual analog scale (VAS; Wang et al., 2020). The VAS for anxiety consisted of a scale ranging from 0 (“no anxiety at all”) to 10 (“highest anxiety level”), which participants rated during each rest period throughout the task.

### 2.5 EEG recording

EEG recording during the experiment involved the utilization of 64 electrode sites in accordance with the international 10–10 system. Two electrodes were placed on the left (M1) and right (M2) ear mastoids as the electrical references, while the anterior frontal zone position (AFz) accommodated the placement of the ground electrode. Moreover, bipolar configurations were established both superior and inferior to the left eye, and on the left and right orbital canthi to record the vertical electrooculogram (VEOU, VEOL) and horizontal electrooculogram (HEOL, HEOR), respectively. The eego system from ANT Neuro (Germany) was deployed for data acquisition, with a bandpass filter set between 1 and 100 Hz and a Notch filter at 50 Hz. Data were collected at a sampling frequency of 500 Hz using eego software, while ensuring that electrode impedance remained below 10 kΩ.

### 2.6 Experimental procedure

The participants were asked not to consume any beverages containing alcohol or caffeine 24 h before testing day. On the testing day, the participants were (a) provided with a brief tour of the testing equipment and apparatus, (b) asked to sign the informed consent form, and (c) given an opportunity to practice football penalty kicking in a practice session to determine their individual task difficulty (a kicking success rate between 40 and 60%) while wearing the Lycra electrode cap to familiarize themselves with the activity. During the session, the target size was adjusted (see Section 2.3.1: Football Penalty Task) to ensure that each participant achieved a kicking success rate between 40 and 60%. Once the appropriate target size was determined, participants were asked to perform three blocks of kicks in the experimental session. Each block comprised 10 kicks, with a 10-minute rest interval between blocks. Throughout the experiment, participants were instructed to focus on the target and kick the ball as accurately as possible. In addition, they were instructed to use their own penalty kicking skills and were allowed to take as many preparatory steps as they normally would before kicking the ball. No additional attentional cues were provided. The entire procedure lasted approximately 90 min to avoid potential fatigue effects on the EEG recordings (Wang et al., 2019, 2021).

### 2.7 Data analysis

#### 2.7.1 EEG data

The EEG data underwent preprocessing using both EEGLAB functions (Delorme and Makeig, 2004) and custom MATLAB scripts (MathWorks, U.S.A.). To preprocess the EEG data, we performed the following steps: (1) re-referenced the data to the averaged mastoids (M1, M2); (2) applied a bandpass filter using a finite impulse response (FIR) filter, ranging from 1 Hz (low-pass) to 30 Hz (high-pass); (3) extracted epochs within a time window of −3,000 to 1,000 ms before the penalty kick; (4) removed channels with bad signal quality; (5) rejected gross artifacts (amplitudes exceeding ± 100 μV) to eliminate any potential biological artifacts (e.g., muscle activation artifacts; Wang et al., 2020), resulting in the rejection of 7 trials in both conditions; (6) performed independent component analysis (ICA; Runica Infomax algorithm; Makeig et al., 1996) to identify and remove components caused by blinks, eye movements, and other non-neural activities; (7) interpolated channels with bad signals; and (8) used random selection for the total number of successful and unsuccessful kicks using random.org. The resulting clean signals were then divided into 2-s epochs spanning a time window of −2,000 to 0 ms before the action. Performance-related power spectrum was defined 8–13 Hz power following Wang et al. (2019). The 8–13 Hz power was computed using the Welch estimation method with a Hanning window function (Welch, 1967). For brevity of reporting, only the results from the key Fz and Cz electrodes, representing the results from their immediate surroundings (i.e., F3, F4, C3, and C4) are presented. These electrodes were selected because they roughly overlie the frontal lobe, which includes the prefrontal cortex, primary motor cortex, premotor cortex, and supplementary motor areas, all of which are related to movement programming processes (i.e., motor planning and motor control) and have been implicated in previous football research (Slutter et al., 2021). For the statistical analysis, a logarithmic (log) transformation of the power values was used to improve normality and reduce variability in the data before conducting further statistical tests.

### 2.8 Statistical analysis

Statistical analyses were performed using SPSS 26.0 (IBM). For EEG data, the Shapiro-Wilk test was used to assess the normality of the transformed data before further statistical analyses. If normality assumptions were violated, Wilcoxon Signed-Rank test was considered. The Shapiro-Wilk test showed no statistically significant deviations from normality for Fz and Cz 8–13 Hz in both successful and unsuccessful performances (*p* > 0.05). Therefore, the data met normality assumptions, allowing for further statistical analyses. Multivariate analyses of variance (MANOVA) for repeated-measures was conducted. Analyses of Fz and Cz were conducted using a 2 (Performance: successful/unsuccessful) in 8–13 Hz repeated-measures MANOVA. *Post hoc* analyses were conducted following significant findings from the ANOVA. Specifically, we utilized the least significant difference (LSD) method and controlled for Type I error using the false discovery rate (FDR) procedure to adjust for multiple comparisons (Genovese et al., 2002). For all statistical tests, including *post hoc* comparisons, the alpha level was set at 0.05 prior to applying FDR corrections. Effect sizes were estimated using the partial η^2^ statistic to quantify the proportion of variance explained by the factors in the ANOVA.

## 3 Results

### 3.1 Parameters of EEG power

**Figure 1:**
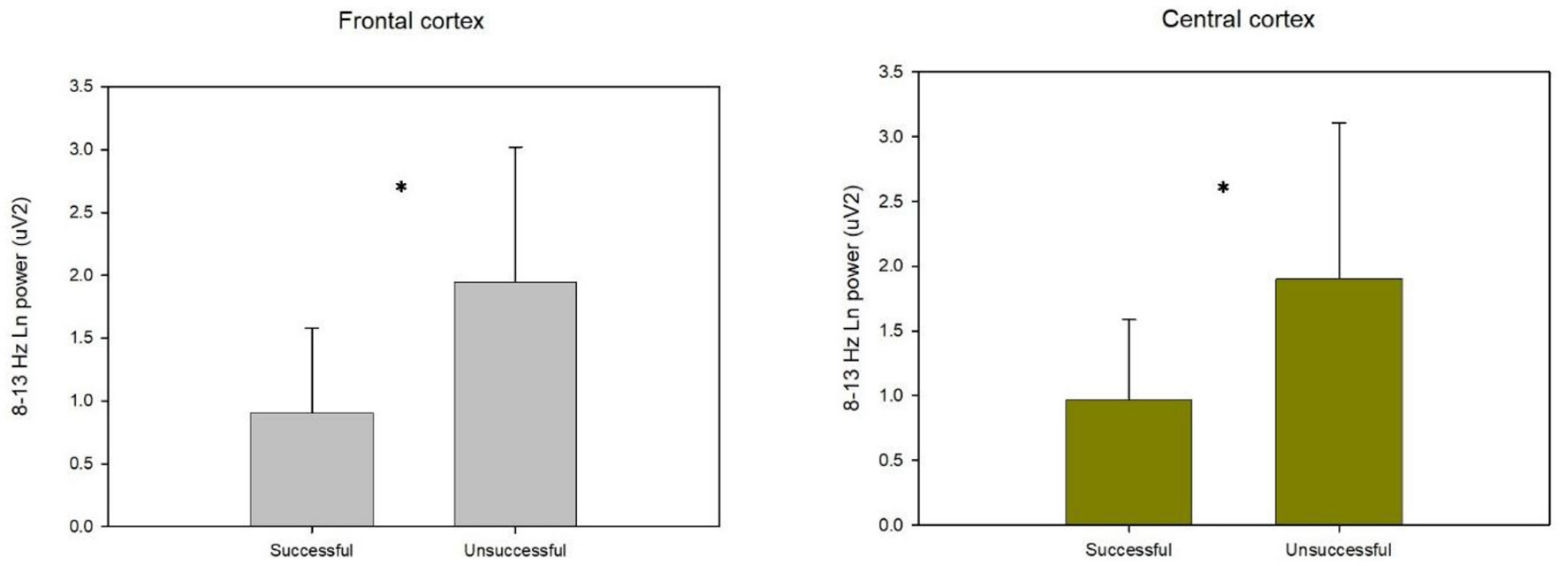
The bar graphs represent the 8–13 Hz a logarithmic (log) transformation of the power values (μV^2^) in the frontal and central cortices during successful and unsuccessful penalty kicks. Error bars indicate standard error of the mean (SEM). *P* < 0.05 indicates a significant difference between successful and unsuccessful kicks. *P < 0.05.

As can be seen in Table 1, a 2 (Performance: successful/unsuccessful) in 8–13 Hz repeated-measures MANOVA of Fz and Cz data revealed a significant interaction effect of performance, *F*_(2, 8)_ = 6.325, *p* = 0.022, Wilks' lambda = 0.386, $\eta_{p}^{2}$ = 0.614. A closer look at the interaction effect indicated a significant effect for the following variables: Fz, *F*_(1, 9)_ = 12.792, *p* = 0.006, $\eta_{p}^{2}$ = 0.587 and Cz, *F*_(1, 9)_ = 4.369, *p* = 0.024, $\eta_{p}^{2}$ = 0.447. *Post hoc* analysis indicated that 8–13 Hz for successful performance was lower than that of unsuccessful performance at Fz (FDR corrected, *p* = 0.012) and Cz (FDR corrected, *p* = 0.024, see Figure 1).

**Table 1:** T-test Results of EEG power associated with successful and unsuccessful performances.

	**Successful performance**		**Unsuccessful performance**			
**EEG**	***M***	***SD***	***M***	***SD***	***t***	***p***
Fz 8–13 Hz	0.90	0.67	1.94	1.07	−3.58	0.012
Mu	0.97	0.61	1.90	1.20	−2.70	0.024

*N* = 10. Mu = Cz 8–13 Hz. Unit: log μV^2^.

### 3.2 Control analyses

#### 3.2.1 Learning effect

To assess whether a learning effect occurred in this study, we compared the success rate across the three blocks using a repeated-measures ANOVA. The results indicated no significant interaction between blocks (*p* = 0.621), suggesting that no learning effect was observed in our study.

#### 3.2.2 VAS anxiety level

The VAS-anxiety level was compared between and within subjects during the football penalty task. A one-way repeated measures ANOVA showed no significant difference between blocks *(p* = 0.257).

## 4 Discussion

This pilot study examined the neuromotor processes during motor preparation for successful performance in the difficult lower limb motor task (i.e., football penalty). Our results mainly showed that successful football penalty was characterized by lower 8–13 Hz power at frontal and central regions. These findings complement previous studies in golf (Babiloni et al., 2008; Cooke et al., 2014; Wang et al., 2019) and football (Slutter et al., 2021) by showing that different types of movement may not modulate EEG power in precision sports (e.g., golf putting and football kicking). Importantly, we further specified the neuromotor processes in adaptive attentional allocation in lower limb motor movement during the difficult task using EEG power analysis.

Successful penalty performance was characterized by lower 8–13 Hz power at frontal and central regions that supports our hypothesis and corresponds with previous golf research. For example, Babiloni et al. (2008), who adopted a 40–60% success rate as a challenging condition, observed that successful performance was associated with more 10–12 Hz ERD in the frontal and central areas. Furthermore, Cooke et al. (2014) and Wang et al. (2019) used EEG power analysis and observed that successful performance was characterized by lower 8–13 Hz power in the frontal and central areas, suggesting that effective allocation of neuromotor processes to these brain regions did not disrupt performance. It is worth noting that 8–13 Hz power in the frontal and central regions reflects cognitive resource allocation in motor programming (Pineda, 2005), such as motor planning (Cooke et al., 2015) and motor control (Wang et al., 2019) during the execution of goal-directed actions (e.g., golf putting). Accordingly, the possible explanation is that the key success in doing penalty kicking in a difficult condition may be associated with mobilizing brain resources for motor planning and motor control before acting (Bertollo et al., 2013; Cooke et al., 2015; di Fronso et al., 2016; Wang et al., 2019). However, our findings extended those of Slutter et al. (2021) by showing that higher activation of the prefrontal cortex was associated with successful penalties in a difficult condition. This inconsistency may be explained by differences in experimental design. Specifically, our study did not include a pressure manipulation. In psychological and motor performance research, pressure manipulation typically involves introducing external stressors, such as social evaluation, competition, and rewards to create a high-pressure environment (Baumeister and Showers, 1986; Cooke et al., 2014). However, our study focused on natural task performance without externally induced pressure-related elements. That is, we controlled this potential moderator factor. Accordingly, we determined that the observed differences brain activity is due to the pressure manipulation in the experiment rather than the types of movement. Taken together, our findings suggest that the EEG-based neuromotor mechanisms specified in golf studies (Babiloni et al., 2008; Cooke et al., 2014; Wang et al., 2019) could be generalized to lower limb movements in sports, such as football kicking.

Overall, the findings of this pilot study suggested that engaging brain resources for motor planning and motor control may be a key element for a successful penalty performance. For example, during a football penalty kick, a player may need to allocate attention to maintain focus on the goal during motor preparation for a successful kick. This process involves activating brain regions responsible for planning and controlling movements. The combined results of our current study enabled us to delineate the fuel characteristic (Wickens and McCarley, 2008) of the attention allocation model and the functional activation of essential neural processes in accordance with the psychomotor efficiency hypothesis (Hatfield, 2018). Furthermore, our findings resonate with the neural proficiency hypothesis of superior performance (Bertollo et al., 2016), which postulates that cognitive control process is essential during motor preparation in difficult situations.

Nevertheless, it is important to acknowledge the limitations of this study. First, the data did not verify whether successful penalty performances included lucky kicks. Future studies should consider measuring the level of certainty of goal achievement for each kick. Second, our study was a pilot study. Although the power analysis by using G^*^Power software indicated that the effect size in our study was 0.89 ($\eta_{p}^{2}$ = 0.447~614), which is a medium effect size according to Cohen's criteria, we still encourage researchers to replicate our study with a larger sample size. Third, a key limitation concerns the volume conduction properties of EEG measurements. Since EEG activity is detected by surface electrodes and primarily reflects activity from near-surface cortical structures, localizing deeper brain sources—especially subcortical networks—remains challenging. This means that observed decreases in activity in specific brain regions may instead reflect a reallocation of resources to other cortical regions or networks that are not directly detectable with EEG. Future studies could address this limitation by employing advanced EEG source localization techniques (e.g., LORETA), combining EEG with complementary neuroimaging methods (e.g., fMRI, fNIRS), or using high-density EEG to improve spatial resolution. Additionally, analyzing functional connectivity rather than single-region activation may provide further insights into the redistribution of neural resources during task execution. Fourth, another potential limitation of this study is attentional strategies among participants. Although we instructed participants to focus on the target and kick the ball as accurately as possible, no specific attentional cues were provided. As a result, individuals may have adopted different focus strategies, which could have influenced their performance and EEG responses. Future studies could consider implementing controlled attentional instructions or monitoring attentional focus to better understand its role in motor performance. Finally, one limitation of this study is that we did not directly measure individual's motivation and intention during experiment, which may be influenced by learning effect and, in turn, affect EEG outcomes. However, no learning effect was observed in the present study, as indicated by the absence of significant differences in success rates across blocks. This suggests that kicking performance remained stable throughout the experiment, potentially minimizing the impact of practice-induced adaptation. Nevertheless, we encourage scholars to replicate this study while assessing the self-report of motivation and intention level.

In summary, the findings of this pilot study provided important insights into the model of attention allocation and psychomotor efficiency hypothesis: selective functional activation of essential neural processes. Specifically, successful football penalty kicks in a difficult task are characterized by lower 8–13 Hz power at frontal and central regions, indicating effective neuromotor allocation without performance disruption. This aligns with similar neuromotor processes in golf studies, highlighting the critical role of motor planning and control in optimizing performance.

## Data Availability

The raw data supporting the conclusions of this article will be made available by the authors, without undue reservation.
